# Information maximization principle explains the emergence of complex cell-like neurons

**DOI:** 10.3389/fncom.2013.00165

**Published:** 2013-11-21

**Authors:** Takuma Tanaka, Kiyohiko Nakamura

**Affiliations:** Department of Computational Intelligence and Systems Science, Interdisciplinary Graduate School of Science and Engineering, Tokyo Institute of TechnologyYokohama, Japan

**Keywords:** information maximization principle, complex cell, primary visual cortex, extraclassical receptive field, computational model

## Abstract

We propose models and a method to qualitatively explain the receptive field properties of complex cells in the primary visual cortex. We apply a learning method based on the information maximization principle in a feedforward network, which comprises an input layer of image patches, simple cell-like first-output-layer neurons, and second-output-layer neurons (Model 1). The information maximization results in the emergence of the complex cell-like receptive field properties in the second-output-layer neurons. After learning, second-output-layer neurons receive connection weights having the same size from two first-output-layer neurons with sign-inverted receptive fields. The second-output-layer neurons replicate the phase invariance and iso-orientation suppression. Furthermore, on the basis of these results, we examine a simplified model showing the emergence of complex cell-like receptive fields (Model 2). We show that after learning, the output neurons of this model exhibit iso-orientation suppression, cross-orientation facilitation, and end stopping, which are similar to those found in complex cells. These properties of model neurons suggest that complex cells in the primary visual cortex become selective to features composed of edges to increase the variability of the output.

## 1. Introduction

A fundamental question that is often raised in neuroscience is how to determine the principle that underlies neural information coding in the brain. In terms of sensory information processing, this question can be answered by explaining how sensory neurons acquire their selectivity to inputs. The primary visual cortex (V1) is an ideal subject for this type of investigation because experimental results regarding the receptive field properties, single-cell electrophysiology, and topographic selectivity map are accumulated in V1. These experimental results allow us to screen the proposed principles of neural information coding by comparing the behavior of the model on the basis of each principle with the receptive field properties of neurons in V1. This screening provides us with a way to deal with the general principle of neural information coding in the cerebral cortex. Several principles having similar mathematical structures, such as the information maximization principle (Linsker, 1988; Bell and Sejnowski, 1997) and sparse coding hypothesis (Barlow, 1959; Olshausen and Field, 1996), were proposed to explain the receptive field properties of simple cells in V1. The statistical independence of the output neurons is the most essential assumption of these models. In the framework of independent component analysis (ICA), the output neurons acquire selectivity to stimuli such that the outputs of these neurons are uncorrelated and as statistically independent as possible. The statistical independence is closely related to the information maximization principle and sparse coding. If the output neurons are statistically dependent, the amount of information conveyed by these neurons reduces because many of them share the same information. Thus, maximizing the amount of information conveyed by the output neurons gives a result similar to that obtained when increasing the statistical independence of the output neurons. If the activity of neurons is independent and uncorrelated, the number of neurons firing simultaneously decreases, and therefore, the neuronal activity becomes sparse.

Since publication of the groundbreaking work by Hubel and Wiesel (1959), it is widely accepted that simple cells have wavelet-like receptive fields, which respond when a wavelet or an edge is positioned appropriately. The ICA models have revealed that ICA of natural images generates output units with simple cell-like receptive field properties. These results suggest that the assumption of the statistical independence of output neurons is a promising principle of neural information coding. However, the ability of this principle to explain the receptive field properties of complex cells has not been addressed. In contrast to simple cells, the response of complex cells is predominantly determined by the orientation of gratings and edges, and these cells are less sensitive to the positions of edges (Hubel and Wiesel, 1962). Thus, it is assumed that complex cells respond to abstract orientation and that the shift-invariant representation of the stimuli in the visual field is accomplished by complex cells. However, recent studies reveal that complex cells are not simple orientation detectors or shift-invariance detectors, and they exhibit surround suppression and facilitation by the gratings outside their classical receptive fields (Jones et al., 2001, 2002). Superimposing gratings perpendicular to the preferred orientation in the receptive field of complex cells suppresses the firing of these cells (Bonds, 1989). These results suggest that the outputs of complex cells are not a simple pooling of simple cell inputs with similar orientation preferences. Using learning principles that can explain the emergence of these complex properties, we will be able to approach the general principle of neural information coding. Conversely, a general principle may shed light on what characterizes the features detected by complex cells. A number of theoretical studies were reported to explain the properties of complex cells (Földiák, 1991; Hyvärinen and Hoyer, 2001; Berkes and Wiskott, 2005; Karklin and Lewicki, 2005, 2009; Shan et al., 2007). Extraclassical receptive fields and complicated receptive field properties were replicated by these studies. However, these models assume that the complex cell-like units receive inputs whose magnitude does not depend on the polarity of the input image pixels (black and white). This type of input facilitates the emergence of the shift-invariant complex cell-like units; however, this assumption should be justified by a general principle.

In this paper, we show that this assumption is justified by using the information maximization principle. In addition, we show that the information maximization principle explains the receptive field properties of complex cells. The differential entropy of the output was used as the measure of information transmitted from the input to the output in the previous ICA models (Bell and Sejnowski, 1995; Shriki et al., 2001). In the Methods section, we introduce mutual information, entropy, and the models we propose in this paper. In the first part of the Results section, we use a three-layer feedforward network comprising an input layer of natural image patches, simple cell-like first-output-layer neurons, and second-output-layer neurons that receive inputs from the first-output-layer neurons (Model 1). Our simulation results obtained using a learning rule that maximizes information transmission show that second-output-layer neurons that receive inputs from first-output-layer neurons with rectifying nonlinearity become shift invariant after the learning process. Some second-output-layer neurons exhibit surround suppression similar to that reported for complex cells. A theoretical calculation based on the fact that the edges detected by simple cells are almost statistically independent proves that the phase insensitivity of complex cells maximizes the output entropy of V1. Model 1 contains realistic first-output-layer neurons with non-negative-definite firing rates. However, Model 1 is computationally expensive so we develop a simplified model of complex cells and more precisely examine the properties of model neurons. In Model 2, the output neurons receive the absolute value of the simple cell-like neurons as inputs. This simplification reduces the computational load and accelerates the convergence. In the Discussion section, we compare our proposed models with previous models and discuss the biological implications of our models.

## 2. Methods

### 2.1. Information maximization principle

Information transmitted from input **x** to output **y** is measured by the mutual information

I(x;y)=H(x)+H(y)−H(x,y),

where *H*(**x**), *H*(**y**), and *H*(**x, y**) are the entropies of the input, the output, and combination of the input and the output. Bell and Sejnowski (1995) showed that the amount of information transmitted from the input layer to the output layer equals the output entropy plus a constant dependent only on the probability distribution of the input. The mutual information can be given by

I(x;y)=H(y)−H(y|x),

where *H*(**y**|**x**) is the entropy of the noise. In the noiseless system, the maximization of the mutual information of input and output can be achieved by the maximization of output entropy. Similar to Bell and Sejnowski (1995), we ignore the intrinsic noise of neurons although it is an important factor contributing to the variability of neuronal firings. We can ignore the effect of the intrinsic noise because qualitatively similar results are obtained by ICA models when we add a small noise to the input and the output of first-output-layer neurons. Thus, by maximizing the output entropy, we expect to have an information-efficient representation of the input in the output layer.

Entropy is a measure of the variability of a random variable, and is defined by

(1) H(x)=−∫p(x)logp(x)dx,

where *p*(**x**) is the probability density function of the random variable **x**. Taking the logarithm to base 2, we have the entropy measured in bits. However, in the following sections, we take logarithms to base *e* because this simplifies the analytical treatment. The entropy of the univariate normal distribution $p(x)=\frac{1}{\sqrt{2\pi \sigma^{2}}}\exp\left(-\frac{{\left(x-\mu \right)}^{2}}{2\sigma^{2}}\right)$, where μ is the mean and σ is the standard deviation, is $\log\sqrt{2\pi e\sigma^{2}}$; the entropy is a monotonically increasing function of the standard deviation. The uniform distribution

(2) $$p(x)=\{\begin{array}{ll} \frac{1}{b-a} & a\leq x\leq b\\ 0 & \text{otherwise} \end{array}$$

has the largest entropy, log(*b* − *a*), among the probability distributions whose domain is between *a* and *b* (Cover and Thomas, 2006). In other words, the uniform distribution is the probability distribution with the highest variability. This means that, under the assumption of rate coding, the entropy of the output of a neuron is large if the maximal firing rate is large and if the firing rate over time is uniformly distributed. The entropy of the *n*-variate normal distribution

(3) $$p(x)=\frac{1}{\sqrt{{\left(2\pi \right)}^{n}\det\Sigma }}\exp\left(-\frac{1}{2}{\left(x-\mu \right)}^{T}\Sigma^{-1}\left(x-\mu \right)\right)$$

is $\log\sqrt{{\left(2\pi e\right)}^{n}\det\Sigma }$, where μ is the mean vector and **Σ** is the covariance matrix. The normal distributions with the mean vectors

(4) $$\mu_{1}=\mu_{2}=\left[\begin{array}{c} \begin{array}{l} 0\\ 0 \end{array} \end{array}\right]$$

and the covariance matrices

(5) $$\Sigma_{1}=\left(\begin{array}{ll} 1 & 0\\ 0 & 1 \end{array}\right)$$

and

(6) $$\Sigma_{2}=\left(\begin{array}{cc} 1 & 1/\sqrt{2}\\ 1/\sqrt{2} & 1 \end{array}\right)$$

have the same marginal distributions, i.e., *x*_1_ and *x*_2_ obey the standard normal distribution in both cases. Thus, the entropies of the marginalized *x*_1_ and *x*_2_ assume the same value for both distributions. However, the joint entropy of the joint distribution of *x*_1_ and *x*_2_ with **Σ**_1_, log_2_(2π*e*) ≈ 4.09 bits, is greater than that of the joint distribution with **Σ**_2_, $\log_{2}\sqrt{{\left(2\pi e\right)}^{2}/2}\approx 3.59$ bits, because the variables *x*_1_ and *x*_2_ are independent in the former and correlated in the latter. Entropy is maximized if the random variables are independent of each other. We can see that the joint entropy of the output of the neurons takes a large value if these neurons fire vigorously and independently. Entropy can therefore be used as a measure of the output variability of a population of neurons. The entropy of the output of neurons in the sensory areas is large if their population firing pattern varies with the input, and it is small if the firing pattern is less affected by the change of input. The output of sensory neurons with large output entropy can be easily utilized by higher sensory areas because different inputs are well separated in the space of the output firing patterns. For this reason, entropy is used as an objective function to train the models of sensory information processing. Another reason for using entropy as an objective function is that entropy is a measure of sensitivity. If the input vector **x** is transformed to the output vector **y** having the same number of elements, the entropy of the output is given by

(7) H(y)=∫p(x)logdetJdx+H(x),

where $J_{ij}=\frac{\partial y_{i}}{\partial x_{j}}$ is the Jacobian matrix of the transform. Because the (*i, j*) entry of the Jacobian matrix is the sensitivity of output *i* to the change of input *j*, the maximization of the output entropy *H*(**y**) can be regarded as the maximization of the sensitivity of output neurons. It is desirable that sensory neurons are maximally sensitive to the change of the stimuli. Note that det **J** cannot always take an arbitrary large value when we use bounded functions, such as in Equation 13, as the activation function of output units.

### 2.2. Model 1

**Figure 1:**
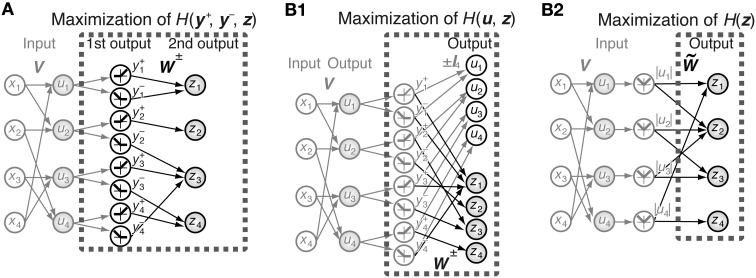
**Schematic representation of the models. (A)** Joint entropy of the first-output-layer **y**^+^ and **y**^−^ and second-output-layer **z** is maximized in Model 1. **V** is the connection weight matrix between *N* = 4 input neurons and 2*N* = 8 first-output-layer neurons, and **W** is the connection weight matrix between the first-output-layer neurons and *N* = 4 second-output-layer neurons. The first-output-layer neurons *y*^+^_*i*_ take on values greater than zero if *u*_*i*_ > 0, and zero otherwise, whereas *y*^−^_*i*_ take on values greater than zero if *u*_*i*_ < 0, and zero otherwise. Black and gray arrows indicate modifiable and fixed connections, respectively. Gray filled circles and open circles are units with and without a nonlinear activation function *f* (*x*), respectively. **(B1)** Entropy of the outputs **u** and **z** is maximized in Model 2. Output *z*_*i*_ is a function of the linear superposition of inputs *y*^+^_*j*_ and *y*^−^_*j*_ by the connection weight matrices **W**^+^ and **W**^−^, respectively. **(B2)** Model 2 is equivalent to the maximization of the entropy of the output **z**, which is a function of the linear superposition of input |*u*_*i*_| by the connection weight matrix $\overset{˜}{W}$.

We assume that the system has an *N*-dimensional input vector **x** and output comprising a 2*N*-dimensional first-output-layer neuron vector **y** = [**y**^+^, **y**^−^] and an *N*-dimensional second-output-layer neuron vector **z** (Figure 1A). The output of the first-output-layer neurons is a deterministic function of input **x** and is described by

(8) $$y_{i}^{+}=R\left(u_{i}\right)$$

and

(9) $$y_{i}^{-}=R\left(-u_{i}\right),$$

where

(10) $$u_{i}=f\left(a_{i}\right)$$

and

(11) $$a_{i}=\sum_{1\leq j\leq N}V_{ij}x_{j}.$$

Here,

(12) $$R(x)=\{\begin{array}{ll} x & x\geq 0\\ 0 & x<0 \end{array}$$

is the ramp function, and

(13) $$f(x)=2\arctan\tanh\frac{x}{2}$$

is the activation function used in this paper. This activation function gives the same algorithm as the ICA algorithm of “tanh nonlinearity” (Hyvärinen et al., 2001) because *f*″(*x*)/*f*′(*x*) = −tanh *x*. This activation function corresponds to the assumption that the independent components follow a sparse distribution *p*(*x*) = 1/(π cosh *x*) in terms of maximal likelihood. Different activation functions corresponding to dense distributions such as that satisfying *f*″(*x*)/*f*′(*x*) = −*x*^3^ cannot predict the receptive field properties of simple cells. Any decomposition matrix of natural images obtained using ICA algorithms can be used as matrix **V** = (*V*_*ij*_). In all simulations of this paper, we use the decomposition matrix obtained by the method described in Section 2.4. The second-output-layer output **z** is a function of the first-output-layer output **y**:

(14) $$z_{i}=f\left(\text{​}-h_{i}\text{​}+\text{​}\sum_{1\leq j\leq N}\text{​}W_{ij}^{+}\text{​}\left(y_{j}^{+}-{\bar{y}}_{j}^{+}\right)+\text{​}\sum_{1\leq j\leq N}W_{ij}^{-}\left(y_{j}^{-}-{\bar{y}}_{j}^{-}\right)\text{​}\right)\text{​},\;$$

where *W*^+^_*ij*_ and *W*^−^_*ij*_ are connection weights, *h*_*i*_ is the offset of the second-output-layer neuron *i*, *ȳ*^+^_*j*_ is the average of *y*^+^_*j*_, and *ȳ*^−^_*j*_ is the average of *y*^−^_*j*_. The terms −*ȳ*^+^_*j*_ and −*ȳ*^−^_*j*_ are introduced to accelerate the convergence. These terms do not affect the values of the parameters after convergence because the increase of *ȳ*^±^_*j*_ to *ȳ*^±^_*j*_ + Δ*ȳ*^±^_*j*_ is offset by the change of *h*_*i*_ to *h*_*i*_ − ∑_1 ≤ *j* ≤ *N*_ *W*^±^_*ij*_ Δ*ȳ*^±^_*j*_.

To enable readers to replicate our results without the need to follow the details of the derivation of the algorithm, here we summarize the simulation process and describe how to evaluate the entropy of the output and derivation of the learning rule in the next section. We use a batch learning process to accelerate the simulation. The weights *W*^+^_*ij*_, *W*^−^_*ij*_, and thresholds *h*_*i*_ are updated once every 100 steps using

(15) $$W^{\pm }\leftarrow W^{\pm }+\epsilon \sum_{1\leq t\leq 100}\Delta W^{\pm }(t)$$

and

(16) $$h\leftarrow h+\epsilon \sum_{1\leq t\leq 100}\Delta h(t),$$

where Δ*W*^±^_*ij*_(*t*) and Δ*h*_*i*_(*t*) are defined by Equations 29 and 32, respectively, and ϵ is the learning rate. The update was performed 1.63 × 10^6^ times with ϵ = 10^−4^ and then 3 × 10^4^ times with ϵ = 10^−5^.

### 2.3. Entropy of output and derivation of the learning rule of Model 1

Here we describe the objective function, the entropy of the output to be maximized, and the derivation of the learning algorithm. First, we define the joint entropy of the first- and second-output-layer neurons, and then we calculate the derivative of the joint entropy with respect to the connection weights. In our model, the *N*-dimensional input vector gives rise to a 2*N*-dimensional first-output-layer vector and an *N*-dimensional second-output-layer vector. The representation is overcomplete because there are more output components than input components. Shriki et al. (2001) considered the maximization of the entropy of the overcomplete representation of the input. Although their model does not contain a multilayer structure and rectifying nonlinearity, the same idea can be applied to Model 1. The probability density of **y** and **z** is related to the input distribution and is given by the relation

(17) $$P(y,z)\propto \frac{P(x)}{\sqrt{\det(\chi^{T}\chi )}},$$

where the susceptibility matrix **χ** ∈ ℝ^3*N* × *N*^ is defined as

(18) $$\chi =\left(\begin{array}{c} Y^{+}\\ Y^{-}\\ Z \end{array}\right).$$

Here we define

(19) $$\begin{array}{l} Y_{ij}^{\pm }=\frac{\partial y_{i}^{\pm }}{\partial x_{j}}\\ \;=\pm s\left(\pm u_{i}\right)f^{\prime }\left(a_{i}\right)V_{ij}, \end{array}$$

(20) $$\begin{array}{l} Z_{ij}=\frac{\partial z_{i}}{\partial x_{j}}\\ \;=f^{\prime }\left(b_{i}\right)\sum_{1\leq k\leq N}\left(W_{ik}^{+}s(u_{k})-W_{ik}^{-}s(-u_{k})\right)f^{\prime }(a_{k})V_{kj}, \end{array}$$

(21) $$b_{i}=-h_{i}+\text{​}\sum_{1\leq j\leq N}\text{​}W_{ij}^{+}\text{​}\left(y_{j}^{+}-{\bar{y}}_{j}^{+}\right)+\text{​}\sum_{1\leq j\leq N}\text{​}W_{ij}^{-}\text{​}\left(\text{​}y_{j}^{-}-{\bar{y}}_{j}^{-}\text{​}\right),\;$$

and

(22) $$s(x)=\{\begin{array}{ll} 1 & x>0\\ \frac{1}{\sqrt{2}} & x=0\\ 0 & x<0 \end{array},$$

because $\frac{\text{d}}{\text{d}x}R(f(x))=s(x)f^{\prime }(x)$ and *f*′(−*x*) = *f*′(*x*). We define $s(0)=1/\sqrt{2}$ to simplify the equation. Here the dependence of **Y** and **Z** on the time step *t* is not explicitly shown. Thus, we maximize the entropy of the output

(23) $$\begin{array}{l} H(y,z)=-\int \int P\left(y,z\right)\log P\left(y,z\right)\text{d}y\text{d}z\\ \;=-\int P\left(x\right)\log\left(\frac{P(x)}{\sqrt{\det(\chi^{T}\chi )}}\right)\text{d}x\\ \;=\frac{1}{2}𝔼\left[\log\det(\chi^{T}\chi )\right]+H(x), \end{array}$$

where 𝔼[·] indicates averaging over the input distribution and we used *P*(**y**, **z**)d**y**d**z** = *P*(**x**)d**x** (Shriki et al., 2001).

The first term of Equation 23 is given by the average of

(24) $$\frac{1}{2}\log\det\left(\chi^{T}\chi \right)=\frac{1}{2}\log\det\left(Y^{+}{}^{T}Y^{+}+Y^{-}{}^{T}Y^{-}+Z^{T}Z\right).\;$$

Here note that

(25) $$\begin{array}{l} {\left(Y^{+}{}^{T}Y^{+}+Y^{-}{}^{T}Y^{-}\right)}_{ij}=\sum_{1\leq k\leq N}V_{ki}\left(s{\left(u_{k}\right)}^{2}f^{\prime }{\left(a_{k}\right)}^{2}+s{\left(-u_{k}\right)}^{2}f^{\prime }{\left(a_{k}\right)}^{2}\right)V_{kj}\\ \;={\left(V^{T}\text{diag}(f^{\prime }{\left(a_{k}\right)}^{2})V\right)}_{ij}, \end{array}$$

where diag(*d*_*k*_) is a diagonal matrix whose diagonal entries are *d*_1_, *d*_2_, …, *d*_*N*_. We also note that

(26) $${\left(Z^{T}Z\right)}_{ij}={\left(V^{T}\text{diag}(f^{\prime }(a_{k}))C^{T}C\text{diag}(f^{\prime }(a_{k}))V\right)}_{ij},$$

where

(27) $$C_{ij}=f^{\prime }(b_{i})\left[W_{ij}^{+}s(u_{j})-W_{ij}^{-}s(-u_{j})\right].$$

From these relations we obtain

(28) $$\begin{array}{l} H(y,z)=\frac{1}{2}\log\det(V^{T}\text{diag}(f^{\prime }{\left(a_{i}\right)}^{2})V+V^{T}\text{diag}(f^{\prime }(a_{i}))\times C^{T}C\text{diag}(f^{\prime }(a_{i}))V)+H(x)\\ \;=\frac{1}{2}\log\det\left(I+C^{T}C\right)+\sum_{1\leq k\leq N}\log f^{\prime }(a_{k})+\log\det V+H(x). \end{array}$$

The second and third terms are the objective function used in the ICA model to generate the connectivity of the first-output-layer neurons (see Section 2.4). The fourth term is the entropy of the input. Differentiating the first term with respect to *W*^±^_*ij*_, we obtain

(29) $$\begin{array}{l} \Delta W_{ij}^{\pm }(t)=\frac{\partial }{\partial W_{ij}^{\pm }}\frac{1}{2}\log\det\left(I+C^{T}C\right)\\ \;=\frac{1}{2}\sum_{1\leq l,m\leq N}{\left[{\left(I+C^{T}C\right)}^{-1}\right]}_{ml}\frac{\partial }{\partial W_{ij}^{\pm }}{\left(C^{T}C\right)}_{lm}\\ \;=\sum_{1\leq l,m\leq N}{\left[{\left(I+C^{T}C\right)}^{-1}\right]}_{ml}\sum_{k}\frac{\partial C_{kl}}{\partial W_{ij}^{\pm }}C_{km}\\ \;=\sum_{1\leq l,m\leq N}{\left[{\left(I+C^{T}C\right)}^{-1}\right]}_{ml}(\pm \delta_{jl}f^{\prime }(b_{i})s(\pm u_{l})\\ \;+\;C_{il}\frac{f^{\prime \prime }(b_{i})}{f^{\prime }(b_{i})}(y_{j}^{\pm }-{\bar{y}}_{j}^{\pm }))C_{im}\\ \;=\pm {\left[C{\left(I+C^{T}C\right)}^{-1}\right]}_{ij}f^{\prime }(b_{i})s(\pm u_{j})\\ \;+{\left[C{\left(I+C^{T}C\right)}^{-1}C^{T}\right]}_{ii}\frac{f^{\prime \prime }(b_{i})}{f^{\prime }(b_{i})}\left(y_{j}^{\pm }-{\bar{y}}_{j}^{\pm }\right), \end{array}$$

where

(30) $$f^{\prime }(x)=\frac{1}{\cosh x}$$

and

(31) $$\frac{f^{\prime \prime }(x)}{f^{\prime }(x)}=-\tanh x.$$

Differentiating the first term of Equation 28 with respect to *h*_*i*_, we obtain

(32) $$\begin{array}{l} \Delta h_{i}(t)=\sum_{1\leq l,m\leq N}{\left[{\left(I+C^{T}C\right)}^{-1}\right]}_{ml}\sum_{1\leq k\leq N}\frac{\partial C_{kl}}{\partial h_{i}}C_{km}\\ \;=-\sum_{1\leq l,m\leq N}{\left[{\left(I+C^{T}C\right)}^{-1}\right]}_{ml}\sum_{1\leq k\leq N}\delta_{ik}C_{kl}\frac{f^{\prime \prime }(b_{k})}{f^{\prime }(b_{k})}C_{km}\\ \;=-{\left[{C(I+C^{T}C)}^{-1}C^{T}\right]}_{ii}\frac{f^{\prime \prime }(b_{i})}{f^{\prime }(b_{i})}. \end{array}$$

Maximization of the entropy of the second output layer, *H*(**z**), can be achieved by replacing (**I** + **C**^*T*^**C**)^−1^ in the above learning rules with (**C**^*T*^**C**)^−1^. Results similar to those presented in this paper are obtained by this modified learning rule, which is equivalent to Model 2 without the constraint *W*^+^_*ij*_ = *W*^−^_*ij*_.

### 2.4. Newton method

We used the Newton method for the ICA model proposed in Amari et al. (1997) and Palmer et al. (2008) to obtain the connection matrix from the input units to first-output-layer neurons and perform the simulation of Model 2. In general, the entropy of real data in ICA models is not a convex function and has multiple local optima. Thus, the global optimum cannot be found using the Newton method in most cases. However, it accelerates the convergence to one of the local optima. The following is the summary of the learning algorithm by Palmer et al. (2008). The entropy of the output is given by the expectation of

(33) $$\log\det V+\sum_{1\leq i\leq N}\log f^{\prime }(y_{i}),$$

where

(34) $$y_{i}=\sum_{1\leq j\leq N}V_{ij}x_{j}.$$

According to their results, the optimal direction Δ*V*_*ij*_(*t*) at step *t* is given by

(35) ΔV(t)=B(t)V,

where

(36) $$B_{ij}=\frac{1+\frac{f^{\prime \prime }(y_{i})}{f^{\prime }(y_{i})}y_{i}}{1+\eta_{i}}\delta_{ij}+\frac{\kappa_{j}\sigma_{i}^{2}\frac{f^{\prime \prime }(y_{i})}{f^{\prime }(y_{i})}y_{j}-y_{i}\frac{f^{\prime \prime }(y_{j})}{f^{\prime }(y_{j})}}{\kappa_{i}\kappa_{j}\sigma_{i}^{2}\sigma_{j}^{2}-1}\left(1-\delta_{ij}\right),$$

(37) $$\kappa_{i}=𝔼\left[\frac{f^{\prime \prime }{\left(y_{i}\right)}^{2}-f^{\prime \prime \prime }(y_{i})f^{\prime }(y_{i})}{f^{\prime }{\left(y_{i}\right)}^{2}}\right],$$

(38) $$\sigma_{i}^{2}=𝔼\left[y_{i}^{2}\right],$$

and

(39) $$\eta_{i}=𝔼\left[\frac{f^{\prime \prime }{\left(y_{i}\right)}^{2}-f^{\prime \prime \prime }(y_{i})f^{\prime }(y_{i})}{f^{\prime }{\left(y_{i}\right)}^{2}}y_{i}^{2}\right],$$

where

(40) $$\frac{f^{\prime \prime }{\left(x\right)}^{2}-f^{\prime \prime \prime }(x)f^{\prime }(x)}{f^{\prime }{\left(x\right)}^{2}}=\frac{1}{\cosh^{2}x}.$$

Here the dependence on *t* is not explicitly shown. For the online algorithm, we updated κ_*i*_, σ^2^_*i*_, and η_*i*_ using

(41) $$\kappa_{i}(t)=\kappa_{i}\left(t-1\right)+\frac{1}{\tau }\left(\frac{1}{\cosh^{2}y_{i}(t)}-\kappa_{i}(t-1)\right),$$

(42) $$\sigma_{i}^{2}(t)=\sigma_{i}^{2}\left(t-1\right)+\frac{1}{\tau }\left(y_{i}{\left(t\right)}^{2}-\sigma_{i}^{2}\left(t-1\right)\right),$$

and

(43) $$\eta_{i}(t)=\eta_{i}(t-1)+\frac{1}{\tau }\left(\frac{y_{i}{\left(t\right)}^{2}}{\cosh^{2}y_{i}(t)}-\eta_{i}(t-1)\right)$$

at each time step. The integration time constant τ was set to be 10, 000 steps in all simulations. The weights *V*_*ij*_ were updated once every 100 steps using

(44) $$V\leftarrow V+\epsilon \sum_{1\leq t\leq 100}\Delta V(t),$$

where ϵ is the learning rate.

The decomposition matrix **V** was obtained using this method. We used randomly selected 20 × 20 pixels image patches from the images and converted the pixels in these image patches to 400-dimensional real-valued inputs, **x**. The mean of the pixels of the image was subtracted from each image. The input images were not prewhitened (Olshausen and Field, 1997) because the input images without the prewhitening process yielded clearer results than the prewhitened ones. The receptive field properties of the second-output-layer neurons obtained using prewhitened images were qualitatively similar to those obtained using non-prewhitened images. The update of the matrix **V** using Equation 44 was performed 10^5^ times with ϵ = 10^−5^, 2.9 × 10^6^ times with ϵ = 10^−4^, and 9.7 × 10^6^ times with ϵ = 10^−5^. The iteration of the learning process of **V** must be sufficiently long as insufficient optimization disrupts the receptive field properties of output neurons.

### 2.5. Model 2

In Model 1, we decomposed *N* pixels into 2*N* first-output-layer values and *N* second-output-layer values. In Model 2, we decomposed 2*N* input values from the rectified linear simple cell-like elements into *N* sign-dependent simple cell-like neurons **u** and *N* complex cell-like neurons **z** (Figure 1B1). In other words, we fixed the output of the first half of the neurons to *u*_*i*_, which are the nonlinear transformations of independent components of the images, and vary the connection weights to the second half of the neurons to obtain complex cell-like properties. 2*N* inputs are given by *y*^+^_*i*_ and *y*^−^_*i*_ and are defined by Equations 8 and 9, respectively. The outputs **u** and **z** are functions of **y** = [**y**^+^, **y**^−^], and are defined as

(45) $$u_{i}=y_{i}^{+}-y_{i}^{-}$$

and

(46) $$z_{i}=f\left(c_{i}\right),$$

where

(47) $$c_{i}=\sum_{1\leq j\leq N}W_{ij}^{+}\left(y_{j}^{+}-{\bar{y}}_{j}^{+}\right)+\sum_{1\leq j\leq N}W_{ij}^{-}\left(y_{j}^{-}-{\bar{y}}_{j}^{-}\right).$$

We do not introduce the offset parameter *h*_*i*_ in this model because *h*_*i*_ takes on values close to zero in the simulation of Model 1. Here we define

(48) $$W=\left(\begin{array}{cc} I_{N} & -I_{N}\\ \begin{array}{c} W^{+} \end{array} & W^{-} \end{array}\right),$$

where **I**_*N*_ is an *N*-dimensional identity matrix. We fix the connection weights of the first half of the neurons, **u**, to simple cell-like receptive fields, and update **W**^+^ and **W**^−^ using an ICA algorithm. Because the covariance matrix of independent components is a diagonal matrix (Hyvärinen et al., 2001), **W**^+^ and **W**^−^ must be appropriately set to decorrelate the outputs. Below, we set **W**^+^ = **W**^−^ = $\overset{˜}{W}$, because assuming *ȳ*^+^_*i*_ = *ȳ*^−^_*i*_, 𝔼[*y*^+^_*i*_ *y*^+^_*j*_] = 𝔼[*y*^−^_*i*_ *y*^−^_*j*_], and 𝔼[*y*^+^_*i*_ *y*^−^_*j*_] = 𝔼[*y*^−^_*i*_ *y*^+^_*j*_], all of which approximately hold for independent components of natural scenes, the covariances 𝔼[*u*_*i*_*c*_*j*_] − 𝔼[*u*_*i*_] 𝔼[*c*_*j*_] vanish only if **W**^+^ = **W**^−^.

Then, the output *z*_*i*_ is given by

(49) $$z_{i}=f\left(\sum_{1\leq j\leq N}{\tilde{W}}_{ij}\left(|u_{j}|-\bar{\left|u_{i}\right|}\right)\right),$$

where |*u*_*i*_| is the average of |*u*_*i*_|, which equals *ȳ*^+^_*j*_ + *ȳ*^−^_*j*_. If input *y*^±^_*i*_ is greater than 0, *y*^∓^_*i*_ is equal to 0; the probability density *p*(*y*^+^_*i*_, *y*^−^_*i*_) is not a function with finite values, and therefore, ICA algorithms cannot be applied. However, assuming that *p*(*y*^+^_*i*_, *y*^−^_*i*_) is a continuous function, the entropy of the output of this system is given by the expectation of

(50) $$\begin{array}{l} \;\log\det W+\sum_{1\leq i\leq N}\log f^{\prime }(c_{i})+H(y)\\ =\log\det\tilde{W}+\sum_{1\leq i\leq N}\log f^{\prime }(c_{i})+N\log2+H(y), \end{array}$$

where the last two terms do not depend on $\overset{˜}{W}$. Hence, the maximization of the first two terms is sufficient for the maximization of the output. This is equivalent to the simulation of the ICA model with the *N*-dimensional input |*u*_*i*_| − |*u*_*i*_| (Figure 1B2). Note that ICA can be applied to it because the probability density of *p*(|*u*_*i*_|) takes finite values, and that the first two terms of Equation 50 equal the first term of Equation 28 if (**I** + **C**^*T*^**C**)^−1^ is replaced by (**C**^*T*^**C**)^−1^ and *W*^+^_*ij*_ = *W*^−^_*ij*_ = ${\tilde{W}}_{ij}$. We perform the Newton method for *N*-dimensional inputs and outputs, which is much faster than the gradient descent of Model 1. The update of the matrix $\overset{˜}{W}$ obtained using Equation 44 was performed 1 × 10^5^ times with ϵ = 10^−6^ and then 2.99 × 10^7^ times with ϵ = 10^−5^.

### 2.6. Characterization of model neurons

**Figure 2:**
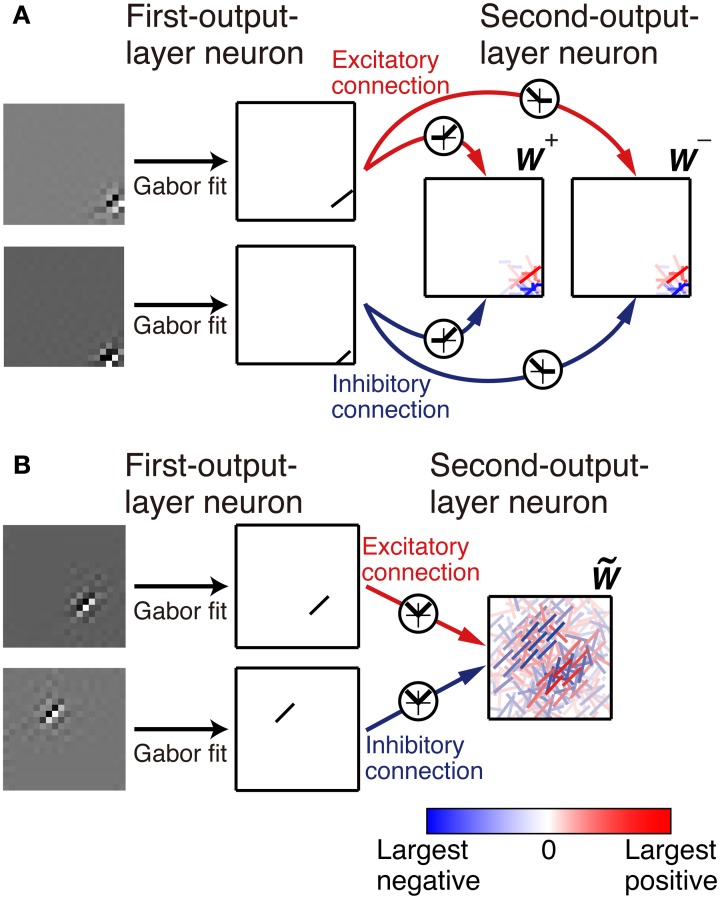
**Representation of the connection weights**. The connection weights from input image patches to the simple cell-like first-output-layer neurons are fitted by a Gabor function. The connection weights from the first-output layer neurons to second-output layer neurons are represented by bars in the second-output-layer neurons. The color and opacity of each bar indicate the sign (red indicates positive, i.e., the excitatory connections, and blue indicates negative, i.e., the inhibitory connections) and magnitude of the connection weight from the first-output-layer neuron, respectively. **(A)**. In Model 1, the left box represents the connection weights from the first-output-layer neurons *y*^+^_1_ through *y*^+^_*N*_, and the right box represents the connection weights from *y*^−^_1_ through *y*^−^_*N*_. **(B)**. In Model 2, connection weights ${\tilde{W}}_{ij}$ are represented by bars in a box.

We fit the connection weights to the first-output-layer neurons from the pixel at (*i, j*) with the Gabor function

(51) $$A\exp\left(-\frac{x^{2}}{2\sigma_{x}^{2}}-\frac{y^{2}}{2\sigma_{y}^{2}}\right)\cos\left(kx-\phi \right)+B,$$

where

$$\begin{array}{l} x=\left(i-x_{0}\right)\cos\theta +\left(j-y_{0}\right)\sin\theta ,\\ y=-\left(i-x_{0}\right)\sin\theta +\left(j-y_{0}\right)\cos\theta \end{array}$$

with parameters *A*, *B*, *x*_0_, *y*_0_, σ_*x*_, σ_*y*_, θ, ϕ, and *k* by using the gradient descent method (Figure 2). The sum of the square of the difference between the fitted function and the connection weights is less than 10% of the sum of the square of the connection weights for 398 out of 400 first-output-layer neurons.

The phase-dependent (F1) to phase-invariant (F0) component ratio (F1/F0 ratio) has been used to characterize simple and complex cells (Shapley and Lennie, 1985; Skottun et al., 1991). Simple cells are identified by the F1/F0 ratio greater than 1, and complex cells are identified by an F1/F0 ratio less than 1. We calculate the F1/F0 ratio of model neuron *i* by using

(52) $$\frac{8}{\pi }\frac{\sqrt{{\left(\sum_{\phi }R\left(z_{i}(\phi )\right)\sin\phi \right)}^{2}+{\left(\sum_{\phi }R\left(z_{i}(\phi )\right)\cos\phi \right)}^{2}}}{\sum_{\phi }R\left(z_{i}(\phi )\right)},$$

where ϕ is the phase of the grating and *z*_*i*_(ϕ) is the output of neuron *i* in response to the grating of the phase ϕ. Under this definition, the F1/F0 ratio equals 2 for the simple cell-like case, *z*_*i*_(ϕ) = *R*(sinϕ), and equals 0 for the perfectly phase-invariant case, *z*_*i*_(ϕ) = 1. We first choose the optimal grating for each neuron and obtain *z*_*i*_(ϕ) by varying the phase of the optimal grating. The optimal grating is chosen from the gratings with various radii (2, 3, 4, 5, and 6 pixels), center positions (*x*, *y* = 1, 2, …, 20), orientations (0°, 20°, …, 340°), spatial frequencies (60°/pixel, 75°/pixel, …, 120°/pixel), and phases (0°, 20°, …, 340°). The center of the optimal grating is used as the center of the gratings when examining the receptive field properties of model neurons (Figures 4, 6).

Neurons tend to adapt to static stimuli and decrease their firing rates. Moving gratings are frequently used to evoke a large response in experiments. Because the response of this model does not depend on the previous stimuli, we use single stationary stimuli as inputs. This does not necessarily mean that the results in this paper correspond to the experimental results obtained using stationary stimuli. We compare the responses of model neurons with experimental results obtained using moving gratings.

## 3. Results

### 3.1. Model 1: information maximization in a three-layer feedforward network

Model 1 is a multilayer feedforward network (Figure 1A). This network contains *N* input units (*x*_*i*_), 2*N* first-output-layer neurons (*y*^+^_*i*_ and *y*^−^_*i*_), and *N* second-output-layer neurons (*z*_*i*_). We used randomly selected 20 pixels × 20 pixels image patches from natural photographs distributed by Prof. Bruno Olshausen on his homepage (Olshausen and Field, 1997) and converted the pixels in these image patches to *N* = 400 real-valued inputs. The input units correspond to the relay neurons in the lateral geniculate nucleus, and first- and second-output-layer neurons correspond to simple and complex cells in V1, respectively. The intensity of a pixel in the input images is represented by a real value in the present paper. In the preprocessing of images, we subtracted the mean pixel intensity from each image; the mean pixel intensity of an image patch is not necessarily zero. First, we performed the learning of the first-output-layer neurons, followed by the learning of the second-output-layer neurons. For first-output-layer neurons to acquire simple cell-like receptive field properties, we set the connection weights from the input units to first-output-layer neurons to the decomposition matrix **V** = (*V*_*ij*_) obtained from the linear ICA of natural image patches. A standard linear ICA algorithm decomposes the *N*-dimensional input vector **x** into an *N*-dimensional independent component vector whose elements can take both signs (Amari et al., 1996). Because the firing rate of a simple cell is not less than zero, we made two first-output-layer neurons *y*^+^_*i*_ and *y*^−^_*i*_ from a non-linear transformation of independent component *i*; if *u*_*i*_ ≥ 0, we set *y*^+^_*i*_ = *u*_*i*_ and *y*^−^_*i*_ = 0, and if *u*_*i*_ < 0, we set *y*^+^_*i*_ = 0 and *y*^−^_*i*_ = −*u*_*i*_, where

(53) $$u_{i}=f\left(\sum_{1\leq j\leq N}V_{ij}x_{j}\right)$$

is the nonlinear transformation of independent component *i* by the sigmoidal activation function *f* (*x*). The first-output-layer neuron *y*^+^_*i*_ is selective to the sign-inversion of the image to which the first-output-layer neuron *y*^−^_*i*_ is selective, and vice versa. The output of the second-output-layer neuron *i* is defined by

(54) $$z_{i}=f\left(-h_{i}+\sum_{1\leq j\leq N}W_{ij}^{+}y_{j}^{+}+\sum_{1\leq j\leq N}W_{ij}^{-}y_{j}^{-}\right),$$

where *W*^+^_*ij*_ and *W*^−^_*ij*_ are the connection weights from the first-output-layer neurons *y*^+^_*j*_ and *y*^−^_*j*_, respectively, and *h*_*i*_ is the threshold. We updated the connection weight matrices **W**^+^ and **W**^−^, and maximized the entropy of the outputs **y**^+^, **y**^−^, and **z**, *H*(**y**^+^,**y**^−^,**z**).

**Figure 3:**
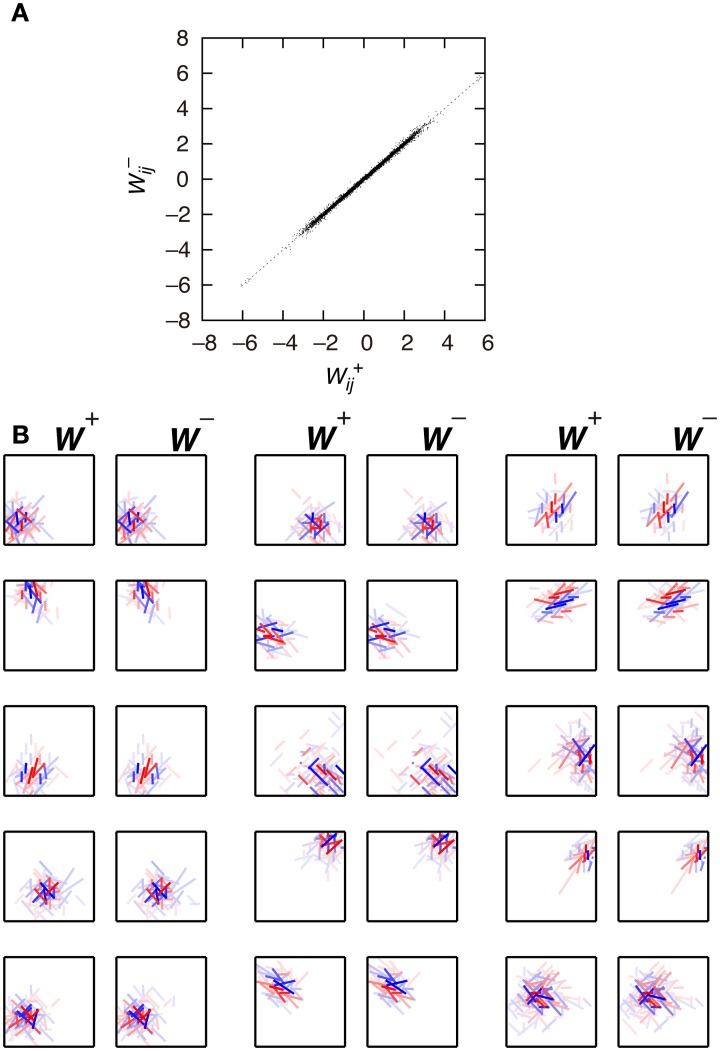
**Connection weights from the first-output-layer neurons to the second-output-layer neurons in Model 1. (A)** Each dot corresponds to the connection weights from two first-output-layer neurons *y*^+^_*j*_ and *y*^−^_*j*_ with sign-inverted receptive fields to the second-output-layer neuron *z*_*i*_. **(B)** Connection weights from the first-output-layer neurons to 15 second-output-layer neurons.

First, we examine the connection weights of the model neurons. Note that we do not impose the constraint *W*^+^_*ij*_ = *W*^−^_*ij*_, which makes second-output-layer neurons invariant to the sign inversion of input images. However, after the learning process, the connection weights *W*^+^_*ij*_ and *W*^−^_*ij*_ result in similar values (Figure 3A, *p* < 0.01, permutation test of Spearman's rank correlation coefficient). To visualize connection weights from first-output-layer neurons to second-output-layer neurons, we have to represent each first-output-layer neuron compactly. We used a Gabor function to fit the connection weights to the first-output-layer neurons, as shown in Figure 2A, and represent the fitted Gabor function with a bar. Thus, the first-output-layer neurons are indicated by bars that represent the optimal orientation and the spatial location of the fitted Gabor function. In the boxes on the right of Figure 2A, we plotted the bars corresponding to the first-output-layer neurons. The left one shows the weights *W*^+^_*ij*_ of a second-output-layer neuron, and the right one shows the weights *W*^−^_*ij*_ of the same neuron. The approximate relation *W*^+^_*ij*_ ≈ *W*^−^_*ij*_ holds in this neuron. Other examples are shown in Figure 3B. The strongest excitatory and inhibitory connections of each neuron are represented by red (RGB[100%, 0%, 0%]) and blue (RGB[0%, 0%, 100%]), respectively. The other connections are represented by a paler red (RGB[100%, 100(1 − *r*)%, 100(1 − *r*)%]) and a paler blue (RGB[100(1 − *r*)%, 100(1 − *r*)%, 100%]), where *r* is the ratio of the strength of the connection to the strongest excitatory or inhibitory connection. These figures indicate that the second-output-layer neurons tend to receive an input having the same sign from pairs of first-output-layer neurons with sign-inverted receptive fields and neurons with similar orientation preferences in a small region.

**Figure 4:**
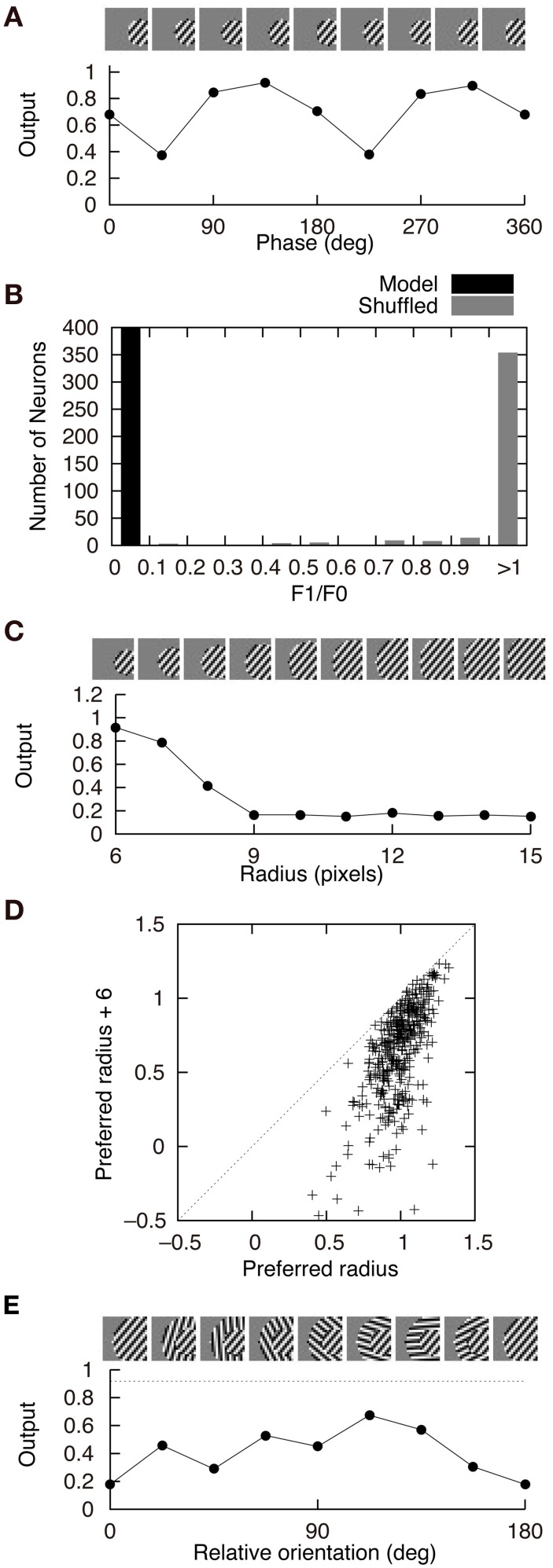
**Properties of second-output-layer neurons in Model 1. (A)** Modulation of the output of the second-output-layer neuron whose connection weights are shown in Figure 2A by the phase of the gratings presented on the top. **(B)** Histogram of the F1/F0 ratio of the model neurons after the learning process (black) and the model neurons whose connection weights are randomly shuffled (gray). **(C)** Change in the output of the same neuron by the radius of the gratings. **(D)** Distribution of the output of 400 second-output-layer neurons in response to the optimal gratings with the preferred radius and to the same gratings whose radius is enlarged by 6 pixels. **(E)** Modulation of the output of the same neuron by the relative orientation of the gratings of the outer annulus to the inner patch. The dashed line indicates the output of the neuron in response to the stimulus with the grating of the inner patch only.

Figure 4 shows the response properties of second-output-layer neurons. The image patches presented to the network as the input are shown on the top of the panels. The output of the neuron in Figure 2A in response to the phase-shifted gratings is shown in Figure 4A. This figure shows that the output is positive for all phases, i.e., this neuron is less sensitive to the phase of the gratings. Insensitivity to the phase of the gratings is a feature of complex cells. Previous experiments have characterized simple and complex cells by measuring the relative modulation or phase-dependent (F1) to phase-invariant (F0) component ratio (F1/F0 ratio) in their responses to the optimal gratings (Shapley and Lennie, 1985; Skottun et al., 1991). Neurons with the F1/F0 ratio greater than 1 are identified as simple cells, whereas those with the F1/F0 ratio less than 1 are identified as complex cells. The F1/F0 ratio of the neuron in Figure 2A is 0.02, which suggests that this cell should be classified as a complex cell. This phase insensitivity is a result of the convergence of the connections having the same sign from two first-output-layer neurons with sign-inverted receptive fields. If a second-output-layer neuron receives connection weights having the same size from each pair of first-output-layer neurons with sign-inverted receptive fields, the sign inversion of the input does not change the output of this second-output-layer neuron. In addition, the convergence of connections having the same sign from the first-output-layer neurons with similar orientation preferences facilitates the phase insensitivity of the second-output-layer neuron. The black bars in Figure 4B represent the histogram of the F1/F0 ratio of second-output-layer neurons. This histogram shows that almost all of them are classified as complex cells. In contrast, most of the model neurons with randomly shuffled connection weights exhibit F1/F0 ratios greater than 1 (Figure 4B, gray bars). Thus, the F1/F0 ratio close to zero is not a result of the multilayer structure because the second-output-layer neurons in the network with random first- to second-output-layer connections do not exhibit the F1/F0 ratio close to zero. On the contrary, this is a result of the information maximization in the multilayer network and the resultant approximate relation *W*^+^_*ij*_ ≈ *W*^−^_*ij*_. These results suggest that the phase insensitivity of complex cells originates from an efficient encoding of the visual input. However, F1/F0 ratios of most model neurons are much smaller than experimentally obtained values. It is reported that a substantial proportion of complex cells have F1/F0 ratios greater than 0.5 (Skottun et al., 1991). This discrepancy may suggest that the simple and complex cells in V1 are not as clearly segregated as the first- and second-output-layer neurons in Model 1.

Stimuli presented in the silent receptive field surrounds can modulate the response of the cells in V1 to the stimuli presented in the classical receptive field (Jones et al., 2002). In most of the cells, the suppression is greatest when the orientation of the gratings in the silent surrounds is the same as the optimal orientation of the classical receptive field. The model neuron shown in Figure 2A is suppressed when the radius of the grating is increased (Figure 4C). Inhibitory connections play an important role in this suppression. Strong inhibitory connections originate from the first-output-layer neurons with similar orientation preferences as the first-output-layer neurons with strong excitatory connections. In this neuron, excitatory and inhibitory areas are separated from each other (Figure 2A). The optimal grating (the leftmost grating of Figure 4C) does not cover the inhibitory area. To systematically examine the suppression of the second-output-layer neurons, we first choose the optimal disk of gratings with the preferred radius for each neuron, and then enlarge them by 6 pixels. Figure 4D shows that enlarging the radius of the optimal gratings decreases the output of most of the second-output-layer neurons. The activity of a second-output-layer neuron is large if the presented grating is restricted to the area of the receptive fields of first-output-layer neurons with excitatory connections, whereas the activity diminishes if the presented grating covers the receptive fields of first-output-layer neurons with inhibitory connections. Jones et al. (2002) reported that iso-orientation suppression was found in 94% of the V1 cells. Similarly, almost all of the second-output-layer neurons (396/400) in Model 1 exhibit the surround suppression. Figure 4E shows the response of the same neuron as in Figure 4C to the surrounding annulus for various orientations in the presence of the inner patch of grating at its preferred orientation. When the orientation of the grating of the outer annulus deviates from the preferred orientation, the degree of suppression diminishes. This type of response, which is classified as iso-orientation suppression (Jones et al., 2002), is a result of the convergence of the excitatory and inhibitory connections from first-output-layer neurons with similar orientation preferences. The first-output-layer neurons with inhibitory connections correspond to the silent surroundings outside the classical receptive field of complex cells. Because the surrounding gratings perpendicular to the center grating suppress the response, this model neuron is classified into the group called “mixed general suppression and orientation alignment suppression” in Jones et al. (2002). The responses to the stimulus with the surrounding grating perpendicular to the center grating are greater than that with the surrounding grating parallel to the center in 376 out of 400 second-output-layer neurons (*p* < 0.01, binomial test). However, the responses to the stimulus with the surrounding grating perpendicular to the center grating are greater than that to the center stimulus alone in only 175 second-output-layer neurons. Jones et al. (2002) reported that 63% of neurons in V1 exhibited cross-orientation facilitation. Model 1 fails to predict the result.

These results suggest that the second-output-layer neurons acquire phase insensitivity and complex cell-like receptive field properties. However, the simulation of Model 1 is computationally expensive, and the learning is very slow to converge. Therefore, we examine the receptive field properties of the neurons in a simplified model.

### 3.2. Model 2: information maximization in a two-layer network with sign-invariant input

Connection weights *W*^+^_*ij*_ and *W*^−^_*ij*_ take on close values in the three-layer network of Model 1 after learning. To examine why this relation holds, we constructed another model network. In Model 2, we maximize the entropy of outputs **u** and **z** (Figure 1B1). Here *u*_*i*_ is identical to the nonlinear transformation of independent component *i* and corresponds to a first-output-layer neuron in Model 1. Output **z** corresponds to second-output-layer neurons in Model 1, and is connected from **y**^+^ and **y**^−^ with connection matrices **W**^+^ and **W**^−^, respectively. We assume that the probability density functions of the outputs of first-output-layer neurons are even functions, and that the signs of these outputs are not correlated. This assumption is supported by the observation that less than 1% of the 2 × 2 contingency tables of the signs of two first-output-layer neurons contain entries that are greater than 0.26 or less than 0.24. Using this assumption, a simple calculation proves that the entropy of the output is maximized if *W*^+^_*ij*_ = *W*^−^_*ij*_ (see Methods). Assuming that *W*^+^_*ij*_ = *W*^−^_*ij*_ = ${\tilde{W}}_{ij}$, Equation 54 leads to

(55) $$z_{i}=f\left(-h_{i}+\sum_{1\leq j\leq N}{\tilde{W}}_{ij}|u_{j}|\right)$$

(Figure 1B2). In this maximization, we use an ICA algorithm with *N* input units, |*u*_*i*_|, and *N* output units, *z*_*i*_. Model 2 reduces the computational load and accelerates the convergence of the learning.

**Figure 5:**
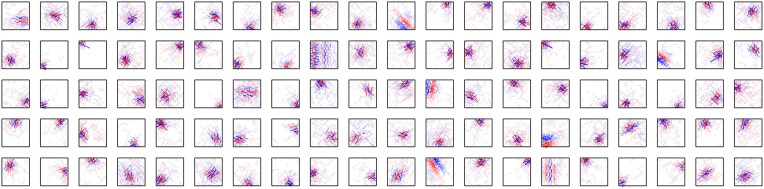
**Connection weights from the input neurons to 100 output neurons in Model 2**.

**Figure 6:**
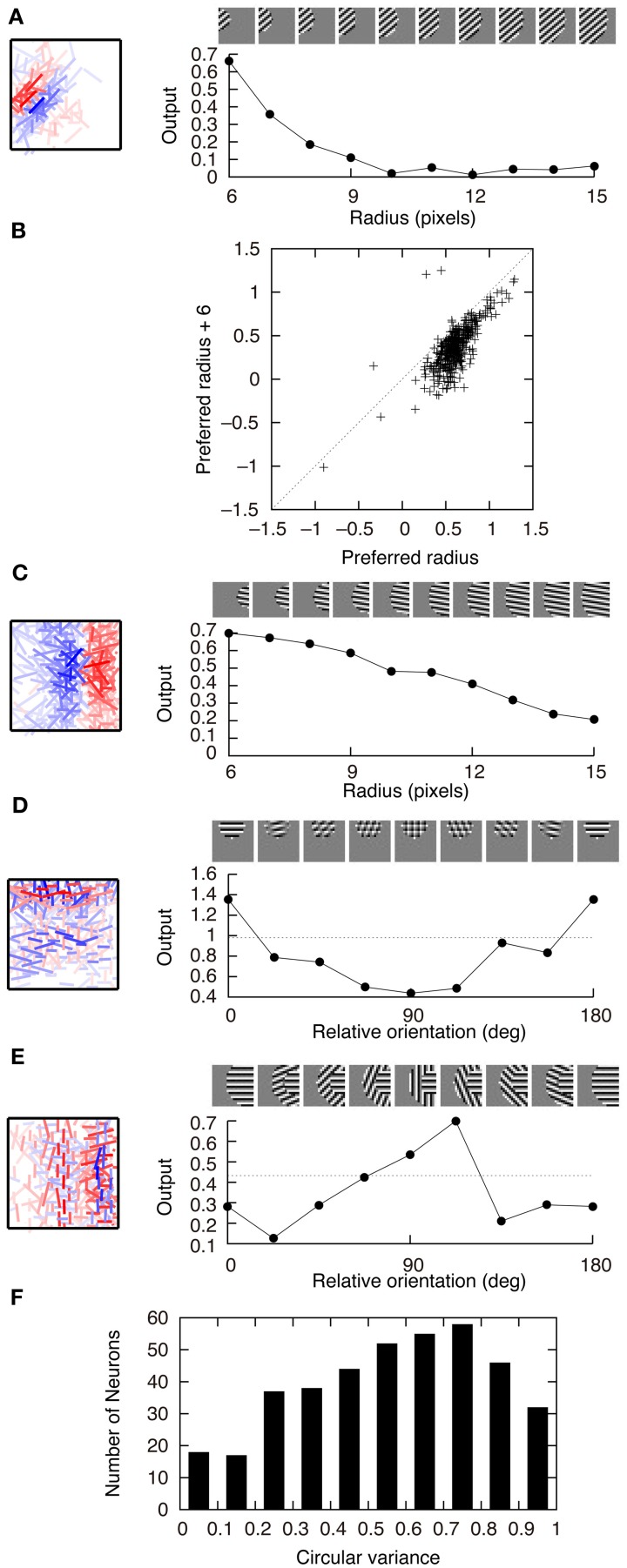
**Properties of output neurons in Model 2. (A)** Change in the output of a neuron by the radius of the gratings. **(B)** Distribution of the output of 400 neurons in response to the optimal gratings with the preferred radius and to the same gratings whose radius is enlarged by 6 pixels. **(C)** End stopping exhibited by a neuron changing its output depending on the radius of the gratings. **(D)** Modification of the output of a neuron by the relative orientation of the gratings superimposed on the optimal grating. The dashed line indicates the output of the neuron in response to the optimal grating only. **(E)** Modulation of the output of a neuron by the orientation of the gratings of the outer annulus. The dashed line indicates the output of the neuron in response to the stimulus with the grating of the inner patch only. **(F)** Histogram of circular variance of orientation tuning of output neurons.

Figure 5 shows the connection weights of 100 output neurons after learning. Because we assume that *W*^+^_*ij*_ = *W*^−^_*ij*_ in this model, the connection weights to a second-output-layer neuron can be represented by a single box (Figure 2B). The inputs to the second output-layer neurons have sparse distributions, whose kurtosis is greater than 3, with the exception of only one model neuron. None of the outputs of first-output-layer neurons, *u*_*i*_, have kurtosis greater than 3. The receptive fields for more than one third of the output neurons are similar to that of the output neuron shown in Figure 2A. Some of them have excitatory and inhibitory connections from input neurons with similar orientation preferences, e.g., the third neuron from the right in the second row. The connection weights from the input neurons with the same orientation selectivity in distant areas of the image patches tend to have inverted signs. Figure 6A shows that this type of neuron is suppressed if a larger grating is presented, in a way similar to that shown in Figure 4C. Figure 6B shows that enlarging the radius of the optimal gratings decreases the output of most neurons. 393 out of 400 neurons of Model 2 also exhibit surround suppression. The neuron in Figure 6C is also suppressed by enlarging the radius of the gratings. However, the orientation preference and spatial alignment of input neurons sending strong inputs to the neuron in Figure 6C are different from those in Figure 6A. The neuron in Figure 6A is suppressed by parallel bars; in contrast, the neuron in Figure 6C is suppressed by a long bar. Thus, the neuron in Figure 6C is an end-stopped neuron (Hubel and Wiesel, 1968), whereas the neuron in Figure 6A is not. Some neurons have much more complex receptive fields and different response properties. Figure 6D shows that the activity of a second-output-layer neuron is suppressed if a grating perpendicular to the preferred orientation of this neuron is superimposed on the center of the receptive field of the neuron. This type of suppression was reported in complex cells (Bonds, 1989). This neuron has excitatory and inhibitory connections from input neurons with orientation selectivity perpendicular to the preferred orientation. In this type of neuron, the connections from the input neurons with orientation selectivity perpendicular to each other in the same area of the image patches tend to have inverted signs. This type of suppression is found in 264 out of 400 second-output-layer neurons (*p* < 0.01, binomial test). Figure 6E shows an example of the response classified as cross-orientation facilitation (Jones et al., 2002). This neuron is suppressed if the radius of the optimal grating is enlarged in a manner similar to that shown in Figures 4C, 6A. When the orientation of the grating of the outer annulus deviates from that of the inner patch, the response exceeds the response to the optimal grating only (shown by the dashed line). In this type of neuron, the connections from the input neurons with orientation selectivity perpendicular to each other in distant areas of the image patches tend to have the same sign. This type of receptive field configuration facilitates the response of the neuron when the orientation of the outer annulus is perpendicular to the preferred orientation of the neuron. This type of facilitation is found in 369 out of 400 second-output-layer neurons (*p* < 0.01, binomial test). The responses to the stimulus with the surrounding grating perpendicular to the center grating are greater than that to the center stimulus alone in 149 second-output-layer neurons only. Model 2 also fails to predict the experimental result of cross-orientation facilitation (Jones et al., 2002). In some other neurons, the orientation preference is unclear (Figure 5). However, these neurons receive strong excitatory and inhibitory connections from input neurons whose preferred positions are restricted to a small area. Thus, these neurons respond selectively to edges in these areas.

Ringach et al. (2002) reported that the circular variance defined by

(56) $$1-\frac{\left|\sum_{\theta }R(z_{i}(\theta ))\exp(2\text{i}\theta )\right|}{\sum_{\theta }R(z_{i}(\theta ))},$$

where θ is the orientation of the grating and *z*_*i*_(θ) is the output of neuron *i* in response to the grating, tends to be greater than 0.5 for complex cells. Figure 6F shows that the circular variance of output neurons of Model 2 tends to be greater than 0.5, which is consistent with experimental results of complex cells.

## 4. Discussion

Our models differ from previous models in several ways. First, to generate sign-insensitive complex cells, Model 1 does not require inputs to be insensitive to the signs of pixels. The model in Hyvärinen and Hoyer (2001) assumes that complex cells receive the square of the output of simple cells. Complex cells in the model by Berkes and Wiskott (2005) are insensitive to the sign of image patches because their output is given by the degree two polynomials of pixel intensities. In the model by Shan et al. (2007), the output of simple cells is transformed to the absolute value and subjected to a nonlinear transformation. The models by Karklin and Lewicki (2005, 2009) also ignore the sign of the output of simple cells because the output of complex cells in these models depends only on the variance of the simple cells. In contrast, Model 1 can generate sign-insensitive complex cells without assuming sign-insensitive inputs to complex cells. The information maximization principle gives a possible explanation as to why sign-insensitive complex cells arise from sign-sensitive simple cell inputs. The results of Model 1 justify the assumption of Model 2 that the output neurons receive the absolute values of the output of simple cell-like neurons as inputs. Second, our models can predict the receptive fields exhibiting surround suppression and facilitation. The complex cell-like receptive fields produced by the models of Földiák (1991) and Hyvärinen and Hoyer (2001) are devoid of these properties. The models of Földiák (1991) and Berkes and Wiskott (2005) also differ from our models in that their models require time-varying sequences of image patches as inputs. Although these models give results similar to our models, none of these models are based on the information maximization principle, but they are based on other optimization criteria such as the parameter fitting of the probability distributions and the minimization of the temporal change of the response.

In Model 2, we transform the *i*-th independent component, *a*_*i*_, to |*u*_*i*_| = |*f* (*a*_*i*_)|. Shan et al. (2007) used a nonlinear function that transforms the absolute value of the independent components into a standard normal distribution. In Model 2, |*u*_*i*_| is almost uniformly distributed rather than normally distributed because the entropy of *f* (*a*_*i*_) is maximized when *f* (*a*_*i*_) is uniformly distributed in the range of the bounded function *f* (*x*). Our results show that a uniformly distributed input can form complex cell-like receptive fields. The model proposed by Karklin and Lewicki (2005) also resembles Model 2. Each output unit of their model detects a specific set of covariances among input variables. For example, an output becomes large when |*x*_1_| and |*x*_2_| are large, and another output becomes large when |*x*_1_| is large and |*x*_2_| is small. The linear superposition of |*u*_*i*_| plays a similar role in Model 2. A large |*u*_*i*_| indicates that the absolute value of the *i*-th simple cell-like input is large, and a small |*u*_*i*_| indicates that the absolute value of the *i*-th simple cell-like input is small. Their model requires the sparseness of the output distribution, which is also required in Model 2, because the linear ICA algorithms can be derived by assuming the sparseness of the source distribution.

The nonlinearity of V1 complex cells was studied by using the models with the pooling of simple cell-like units (Sakai and Tanaka, 2000; Martinez and Alonso, 2001). We attempted to examine the second-order nonlinearity of the second-output-layer neurons. However, reverse correlation of these neurons did not exhibit second-order Wiener-like kernels that are similar to those observed for complex cells (Szulborski and Palmer, 1990). This is presumably because the widths of on- and off-regions of most first-output-layer neurons are as narrow as 1 pixel.

From our study, we speculate that complex features can be detected by combining the inputs from a large number of simpler elements. By increasing the number of output layers in our models, we will have higher-order neurons that are selective to more complex features. The information maximization of a multilayer network with higher-order neurons would be capable of explaining the complicated selectivity of neurons in higher visual areas.

However, there are some limitations to our models, such as the fact that our models use feedforward networks. Although this structure simplifies the models and facilitates the derivation of the learning rules, cortical networks have feedback and recurrent structures as well as feedforward structures. It is known that the feedback from higher to lower sensory areas plays an essential role in sensory cortices. Bardy et al. (2006) reported that the inactivation of the feedback from the higher visual areas affected the selectivity of the neurons in V1. They found that this inactivation changed the responses of a substantial proportion of neurons classified as complex cells to simple cell-like responses, indicating that the feedback from higher visual areas modifies the receptive fields of complex cells. The response of complex cells appears to be formed by both a feedforward mechanism and a feedback and recurrent mechanism. Simple cells, which are assumed to provide inputs to complex cells in this paper, receive recurrent connections so that they exhibit surround suppression (Burr et al., 1981; Walker et al., 2000). Ringach et al. (2002) showed that simple cells with odd-symmetric receptive fields in V1 are greater in number than those predicted from ICA models. This may be the reason for which reverse correlation of the second-output-layer neurons did not exhibit Wiener-like kernels that are similar to those observed for complex cells. Although the receptive fields of some second-output-layer neurons in our models are irregular and different from model neurons with typical complex cell-like receptive fields, the introduction of higher-order neurons and recurrent connections (Tanaka et al., 2009) could cause the first-output-layer neurons to exhibit properties that are more similar to simple cells; in turn, it may increase the number of model neurons with complex cell-like receptive fields.

### Conflict of interest statement

The authors declare that the research was conducted in the absence of any commercial or financial relationships that could be construed as a potential conflict of interest.
